# Hyperglycemic Condition Causes Pro-Inflammatory and Permeability Alterations Associated with Monocyte Recruitment and Deregulated NFκB/PPARγ Pathways on Cerebral Endothelial Cells: Evidence for Polyphenols Uptake and Protective Effect

**DOI:** 10.3390/ijms22031385

**Published:** 2021-01-30

**Authors:** Janice Taïlé, Jessica Patché, Bryan Veeren, Marie-Paule Gonthier

**Affiliations:** Diabète athérothrombose Thérapies Réunion Océan Indien, INSERM, UMR 1188, Université de La Réunion, 2 rue Maxime Rivière, 97490 Sainte-Clotilde, La Réunion, France; janice.taile@univ-reunion.fr (J.T.); jessica.patche@univ-reunion.fr (J.P.); bryan.veeren@univ-reunion.fr (B.V.)

**Keywords:** cerebral endothelial cell, hyperglycemia, inflammation, permeability, polyphenols, medicinal plants

## Abstract

Hyperglycemia alters the function of cerebral endothelial cells from the blood-brain barrier, increasing the risk of cerebrovascular complications during diabetes. This study evaluated the protective effect of polyphenols on inflammatory and permeability markers on bEnd3 cerebral endothelial cells exposed to high glucose concentration. Results show that hyperglycemic condition increased nuclear factor kappa B (NFκB) activity, deregulated the expression of interleukin-1 beta (*IL-1β*), interleukin-6 (*IL-6*), tumor necrosis factor-alpha (*TNF-α*), cyclooxygenase-2 (*COX-2*), inducible nitric oxide synthase (*iNOS*), interleukin-10 (*IL-10*) and endothelial-leukocyte adhesion molecule (*E-selectin*) genes, raised MCP-1 secretion and elevated monocyte adhesion and transendothelial migration. High glucose decreased occludin, claudin-5, zona occludens-1 (ZO-1) and zona occludens-2 (ZO-2) tight junctions production and altered the endothelial permeability. Characterized polyphenolic extracts from the French medicinal plants *Antirhea borbonica*, *Ayapana triplinervis*, *Dodonaea viscosa* and *Terminalia bentzoe*, and their major polyphenols quercetin, caffeic, chlorogenic and gallic acids limited the pro-inflammatory and permeability alterations caused by high glucose. Peroxisome proliferator-activated receptor gamma (PPARγ) agonist also attenuated these damages while PPARγ antagonist aggravated them, suggesting PPARγ protective action. Interestingly, polyphenols improved *PPARγ* gene expression lowered by high glucose. Moreover, polyphenols were detected at the intracellular level or membrane-bound to cells, with evidence for breast cancer resistance protein (BCRP) efflux transporter role. Altogether, these findings emphasize the ability of polyphenols to protect cerebral endothelial cells in hyperglycemic condition and their relevance for pharmacological strategies aiming to limit cerebrovascular disorders in diabetes.

## 1. Introduction

The blood-brain barrier (BBB) is a crucial cellular barrier responsible for a strict regulation of paracellular permeability between the blood and the brain tissue [1,2]. Microvascular cerebral endothelial cells which compose the BBB are interconnected by tight junctions constituted with three major transmembrane proteins which are occludins, claudins and junctional adhesion proteins. Other tight junction proteins such as zona occludens-1 (ZO-1) and zona occludens-2 (ZO-2) are important for connecting transmembrane proteins with the cytoskeleton at the intracellular level. These multiprotein junctional complexes are essential to regulate paracellular permeability in order to maintain BBB tightness, and their production is strictly regulated via signaling pathways involving key transcriptional factors such as the nuclear factor kappa B (NFκB) and the peroxisome proliferator-activated receptor gamma (PPARγ) [3,4,5,6,7]. Cerebral endothelial cells also express various families of drug efflux transport proteins and particularly two major efflux transporters which are the P-glycoprotein (Pgp) and the breast cancer resistance protein (BCRP) that restrict substances penetrating the brain [8]. Indeed, these extra- and intracellular components limit permeability to low molecular mass molecules and increase barrier electrical resistance. Of note, gelatinolytic matrix metalloproteinases (MMP) and particularly MMP-2 and MMP-9 play a major role in various disorders and mainly in diabetes cases via the degradation of intercellular junction proteins, leading to BBB disruption [9,10]. Furthermore, it is well known that during type 2 diabetes, the hyperglycemic condition leads to vascular endothelial inflammation through the activation of NFκB which increases the production of pro-inflammatory cytokines including tumor necrosis factor-alpha (TNF-α), interleukins like interleukin-1 beta (IL-1β) and interleukin-6 (IL-6) as well as monocyte chemoattractant protein-1 (MCP-1) [11]. Moreover, hyperglycemia-associated inflammation induces an overproduction of cell adhesion molecules in endothelial cell surface such as endothelial-leukocyte adhesion molecule (E-selectin) promoting the adherence and the transendothelial migration of circulating monocytes and other inflammatory cells through the BBB [12,13]. Additionally, pro-inflammatory cytokine secretion leads to a reduction in Pgp and BCRP expression and activities [14]. Taken together, these vascular endothelial dysfunctions lead to the BBB impairment. 

Plant polyphenols are the most abundant antioxidants provided by the human diet. Among them, flavonoids such as quercetin and phenolic acids like caffeic, chlorogenic and gallic acids are the most consumed [15]. Several studies reported that dietary polyphenols exert beneficial health effects through metabolic and anti-inflammatory actions that target the endothelium and improve the vascular function [16,17,18,19]. Polyphenols also constitute the active substances present in many medicinal plants. Recently, we showed that polyphenols extracted from four medicinal plants referenced in the French Pharmacopeia for antidiabetic properties, namely *Antirhea borbonica*, *Ayapana triplinervis*, *Dodonaea viscosa* and *Terminalia bentzoe*, attenuate oxidative stress and the alteration of vasoactive markers production in cerebral endothelial cells during the hyperglycemic condition. Quercetin, caffeic, chlorogenic and gallic acids that we detected as major polyphenols in these medicinal plant extracts exhibit similar protective properties [20,21]. However, the impact of these polyphenolic plant extracts on the inflammatory response and permeability of cerebral endothelial cells remains poorly elucidated. 

The present study aimed to determine the protective effects of medicinal plant polyphenols on inflammatory and permeability markers as well as monocyte recruitment on cerebral endothelial cells exposed to hyperglycemic condition. The characterized polyphenol-rich extracts from *A. borbonica*, *A. triplinervis*, *D. viscosa* and *T. bentzoe* plants, and pure quercetin, caffeic, chlorogenic and gallic acids identified as predominant phytochemicals were tested. In an innovative way, the uptake of polyphenols by cerebral endothelial cells was evaluated by comparing their intracellular and membrane-bound levels, in the presence or not of specific inhibitors targeting Pgp and BCRP efflux transporters.

## 2. Results

**Figure 1:**
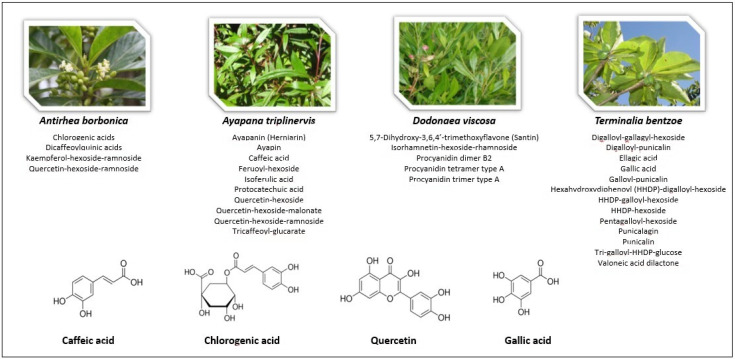
Polyphenol-rich medicinal plant extracts and pure polyphenols tested. The polyphenols present in the extracts obtained from *A. borbonica*, *A. triplinervis*, *D. viscosa* and *T. bentzoe* medicinal plants were previously identified [21]. Caffeic acid, chlorogenic acid and quercetin derivatives were detected as main polyphenols present in *A. borbonica*, *A. triplinervis* and *D. viscosa* plants, while gallic acid derivatives were detected in *T. bentzoe* plant. Consistently, pure quercetin, caffeic, chlorogenic and gallic acids were used as control polyphenols in the experiments.

The composition of the characterized polyphenol-rich extracts from *A. borbonica*, *A. triplinervis*, *D. viscosa* and *T. bentzoe* plants, as well as the polyphenols quercetin, caffeic, chlorogenic and gallic acids used for the experiments are presented on Figure 1.

### 2.1. Effect of Hyperglycemic Condition and Polyphenols on the Production of Inflammatory Markers on Cerebral Endothelial Cells

**Figure 2:**
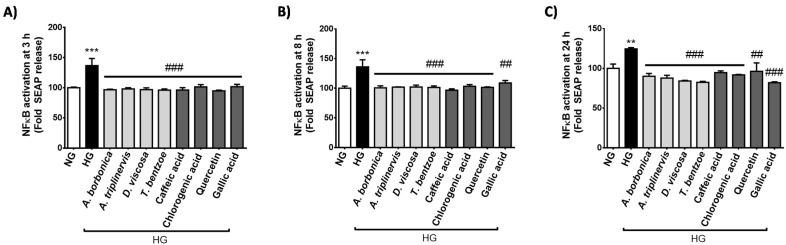
Effect of hyperglycemic condition and polyphenols on the transcriptional activity of NFκB in cerebral endothelial cells. Cells were exposed to normoglycemic condition (NG) or hyperglycemic condition (HG) in the presence or not of each polyphenol-rich plant extract (10 μM GAE) or a pure polyphenol (10 μM). Relative NFκB/SEAP activity was determined by Quanti-Blue assay at 3 h (**A**), 8 h (**B**) and 24 h (**C**) and expressed as the percentage of the control NG condition. Data are means ± SEM of three independent experiments (three cellular passages). **: *p* <0.01 and ***: *p* <0.005 as compared to NG; ^##^: *p* <0.01 and ^###^: *p* <0.005 as compared to HG.

During diabetes, hyperglycemia may mediate pro-inflammatory effects via the activation of the transcriptional factor NFκB that regulates the expression of genes coding for cytokines. We evaluated NFκB transcriptional activity in cells exposed to normoglycemic or hyperglycemic condition, in the presence or not of polyphenols for 3 h (Figure 2A), 8 h (Figure 2B) and 24 h (Figure 2C). Results show that high glucose condition significantly elevated the transcriptional activity of NFκB from 3–24 h. All polyphenol-rich plant extracts and pure polyphenols counteracted the increase in NFκB activity mediated by hyperglycemic condition. 

**Figure 3:**
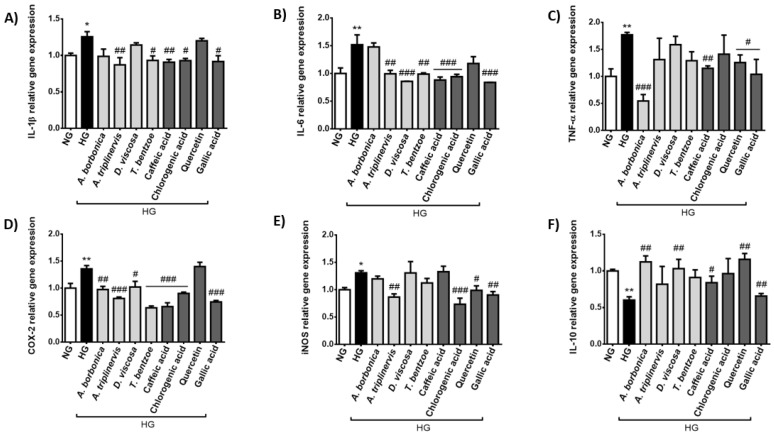
Effect of hyperglycemic condition and polyphenols on the inflammatory response of cerebral endothelial cells. Cells were exposed to normoglycemic condition (NG) or hyperglycemic condition (HG) in presence or not of each polyphenol-rich plant extract (10 μM GAE) or a pure polyphenol (10 μM). Relative expression of genes coding for IL-1β (**A**), IL-6 (**B**), TNF-α (**C**), COX-2 (**D**), iNOS (**E**) and IL-10 (**F**) was measured by RT-qPCR and normalized to *GAPDH* gene expression. Data are means ± SEM of three independent experiments (three cellular passages). *: *p* < 0.05 and **: *p* < 0.01 as compared to NG; ^#^: *p* < 0.05, ^##^: *p* < 0.01 and ^###^: *p* < 0.005 as compared to HG.

Concordantly, the results from RT-qPCR analysis indicate that hyperglycemic condition led to an up-regulated expression of the genes encoding the pro-inflammatory markers IL-1β (Figure 3A), IL-6 (Figure 3B), TNF-α (Figure 3C), COX-2 (Figure 3D) and iNOS (Figure 3E). Conversely, high glucose condition down-regulated the expression of the gene coding for the anti-inflammatory cytokine IL-10 (Figure 3F). *A. borbonica* extract reduced *TNF-α* and *COX-2* gene expression while it raised *IL-10* gene expression. *A. triplinervis* extract decreased *IL-1β*, *IL-6*, *COX-2* and *iNOS* gene expression. *D. viscosa* extract attenuated *IL-6* and *COX-2* gene expression, whereas it improved *IL-10* gene expression. *T. bentzoe* extract led to a reduction in *IL-1β*, *IL-6* and *COX-2* gene expression. Gallic acid improved the inflammatory response deregulated by high glucose, by decreasing the production of all the pro-inflammatory markers studied and by increasing that of IL-10. Caffeic acid also modulated the expression of genes coding for all markers, except for iNOS. Chlorogenic acid reduced the expression of *IL-1β*, *IL-6*, *COX-2* and *iNOS* genes. Quercetin lowered the expression of *TNF-α* and *iNOS* genes, and elevated that of *IL-10* gene. 

**Figure 4:**
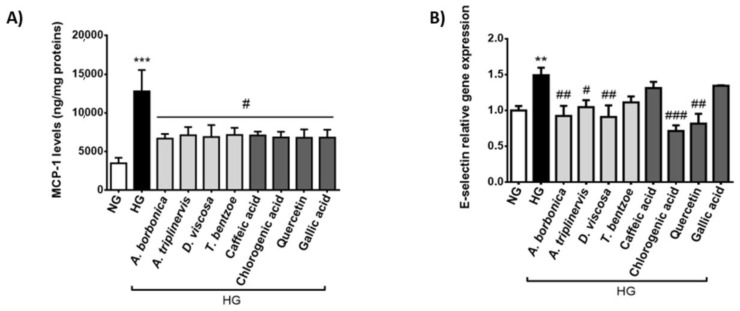
Effect of hyperglycemic condition and polyphenols on markers involved in endothelial cell and leukocyte interaction. Cells were exposed to normoglycemic condition (NG) or hyperglycemic condition (HG) in presence or not of each polyphenol-rich plant extract (10 μM GAE) or a pure polyphenol (10 μM). The secretion of MCP-1 was measured by ELISA kit (**A**). The expression of *E-selectin* gene was measured by RT-qPCR and normalized to *GAPDH* gene expression (**B**). Data are means ± SEM of three independent experiments (three cellular passages). **: *p* < 0.01 and ***: *p* < 0.005 as compared to NG; ^#^: *p* < 0.05, ^##^: *p* < 0.01 and ^###^: *p* < 0.005 as compared to HG.

In order to assess the effect of the hyperglycemic condition and polyphenols on the production of inflammatory markers involved in endothelial cell and leukocyte interaction, MCP-1 secreted levels (Figure 4A) and *E-selectin* gene expression rate (Figure 4B) were measured. We found that hyperglycemic condition induced a 3-fold increase in MCP-1 release. All polyphenol-rich plant extracts and pure polyphenols significantly decreased MCP-1 secretion mediated by hyperglycemic condition. High glucose also led to an elevation of E-selectin gene expression while *A. borbonica*, *A. triplinervis* and *D. viscosa* extracts as well as chlorogenic acid and quercetin protected against this deleterious action of hyperglycemic condition. Collectively, these data suggest the ability of polyphenol-rich plant extracts and pure polyphenols to attenuate the pro-inflammatory response of cerebral endothelial cells caused by hyperglycemic condition. The extent of the anti-inflammatory action of polyphenols depended on the nature of the plant extract, phenolic compound and inflammatory marker considered. 

### 2.2. Effect of Hyperglycemic Condition and Polyphenols on the Monocyte Adhesion and Transendothelial Migration 

**Figure 5:**
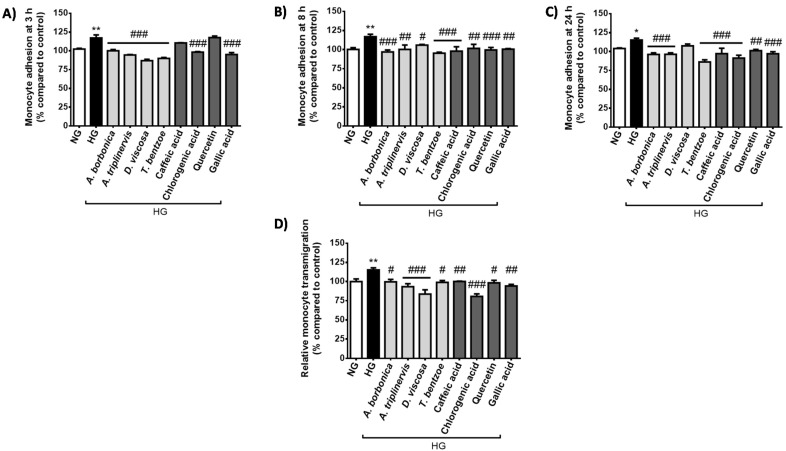
Effect of hyperglycemic condition and polyphenols on monocyte adhesion and transendothelial migration. Cells were exposed to normoglycemic condition (NG) or hyperglycemic condition (HG) in presence or not of each polyphenol-rich plant extract (10 μM GAE) or a pure polyphenol (10 μM). Relative adhesion of THP-1 cells on bEnd3 cerebral endothelial cells was measured at 3 h (**A**), 8 h (**B**) and 24 h (**C**) and expressed as the percentage of the control NG condition. Relative transendothelial migration of THP-1 cells through bEnd3 cellular barrier was determined at 24 h (**D**) and expressed as the percentage of the control NG condition. Data are means ± SEM of three independent experiments (three cellular passages). *: *p* < 0.05 and **: *p* < 0.01 as compared to NG; ^#^: *p* < 0.05, ^##^: *p* < 0.01 and ^###^: *p* < 0.005 as compared to HG.

Hyperglycemia is reported to promote the adhesion of monocytes on cerebral endothelial cells, leading to monocyte transendothelial migration. We determined the effect of polyphenols on THP-1 monocyte adhesion on bEnd3 cerebral endothelial cell during high glucose exposure of 3 h (Figure 5A), 8 h (Figure 5B) and 24 h (Figure 5C). The results demonstrate that high glucose led to a significant elevation of monocyte adhesion from 3–24 h. Polyphenols exerted a protective effect by decreasing monocyte adhesion induced by hyperglycemic condition. Noticeably, polyphenol action was dependent on the nature of the medicinal plant or phenolic compound tested and was also time-dependent. Indeed, polyphenol-rich extract from *D. viscosa* reduced monocyte adhesion from 3–8 h but was inefficient at 24 h, in contrast to the three other plant extracts which exhibited protective properties from 3–24 h similarly to chlorogenic and gallic acids. While caffeic acid and quercetin did not modulate monocyte adhesion at 3 h, they were able to attenuate it from 8–24 h, underlining the possibility for different cellular accessibility rates or mechanisms of action. In parallel, we found that the monocyte transendothelial migration was significantly increased during a 24-h high glucose exposure (Figure 5D). All polyphenol-rich plant extracts and pure polyphenols exerted a protective role by limiting the monocyte transendothelial migration mediated by hyperglycemic condition. 

### 2.3. Effect of Hyperglycemic Condition and Polyphenols on the Production of Permeability Markers on Cerebral Endothelial Cells 

**Figure 6:**
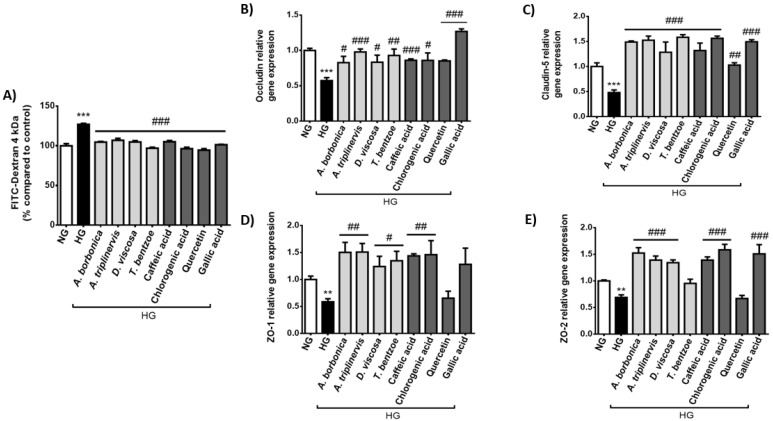
Effect of hyperglycemic condition and polyphenols on the permeability and the expression of genes coding tight junction proteins in cerebral endothelial cells. Cells were exposed to normoglycemic condition (NG) or hyperglycemic condition (HG) in presence or not of each polyphenol-rich plant extract (10 μM GAE) or a pure polyphenol (10 μM). Relative endothelial permeability (**A**) was determined through FITC-Dextran assay and expressed as the percentage of the control NG condition. Relative expression of genes coding for occludin (**B**), claudin-5 (**C**), ZO-1 (**D**) and ZO-2 (**E**) was measured by RT-qPCR and normalized to *GAPDH* gene expression. Data are means ± SEM of three independent experiments (three cellular passages). **: *p* < 0.01 and ***: *p* < 0.005 as compared to NG; ^#^: *p* < 0.05, ^##^: *p* < 0.01 and ^###^: *p* < 0.005 as compared to HG.

The tight junctions occludin, claudin-5, ZO-1 and ZO-2 play a critical role for maintaining the restrictive paracellular permeability of the BBB. We assessed the impact of high glucose concentration on the endothelial permeability to FITC-Dextran and the expression of genes encoding tight junctions. The results show that the hyperglycemic condition led to an increased FITC-Dextran permeability reaching 127.01 ± 1.30% (Figure 6A). Moreover, RT-qPCR data demonstrate that high glucose caused a down-regulation of the expression of genes coding for occludin (Figure 6B), claudin-5 (Figure 6C), ZO-1 (Figure 6D) and ZO-2 (Figure 6E). Importantly, all polyphenol-rich plant extracts and pure polyphenols counteracted the deleterious effect of hyperglycemic condition on FITC-Dextran permeability and *claudin-5* gene expression. Regarding *occludin* gene expression, all polyphenols tested exerted a protective action, except quercetin. Concerning *ZO-1* gene expression, polyphenol-rich plant extracts, caffeic and chlorogenic acids counteracted the high glucose effect. For *ZO-2* gene expression, all plant extracts except *T. bentzoe* extract and the pure compounds caffeic, chlorogenic and gallic acids reversed the action of hyperglycemic condition. Thus, here again the extent of the protective action of polyphenols was dependent on the nature of the plant extract, phenolic compound and tight junction marker tested. 

### 2.4. Effect of PPARγ agonist and Antagonist on the Inflammatory and Permeability Alterations Caused by Hyperglycemic Condition on Cerebral Endothelial Cells

**Figure 7:**
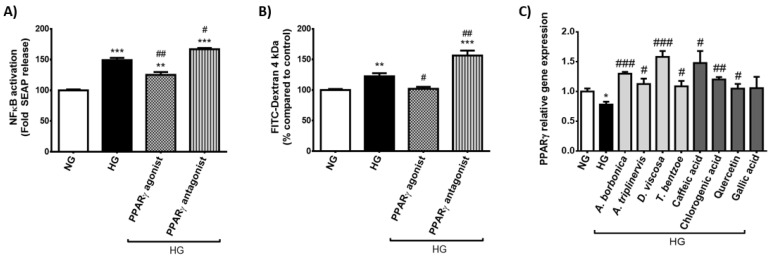
Effect of PPARγ agonist and antagonist on the inflammatory and permeability alterations caused by hyperglycemic condition in cerebral endothelial cells. Cells were exposed to normoglycemic condition (NG) or hyperglycemic condition (HG) in presence or not of PPARγ agonist or antagonist (10 μM) for 3 h. Relative NFκB/SEAP activity (**A**) and relative endothelial permeability (**B**) were determined by Quanti-Blue and FITC-dextran assays, respectively, and expressed as the percentage of the control NG condition. In parallel, cells were exposed to normoglycemic condition (NG) or hyperglycemic condition (HG) in presence or not of each polyphenol-rich plant extract (10 μM GAE) or a pure polyphenol (10 μM) for 3 h. Relative expression of *PPARγ* gene was measured by RT-qPCR and normalized to *GAPDH* gene expression (**C**). Data are means ± SEM of three independent experiments (three cellular passages). *: *p* < 0.05, **: *p* < 0.01 and ***: *p* < 0.005 as compared to NG; ^#^: *p* < 0.05, ^##^: *p* < 0.01 and ^###^: *p* < 0.005 as compared to HG.

The transcriptional factor PPARγ has been reported to protect the BBB integrity via improvement of tight junction proteins. We assessed the effect of PPARγ agonist (Pioglitazone) or antagonist (GW9662) on markers of the inflammatory response and permeability changes induced by hyperglycemic condition, by measuring NFκB transcriptional activity and FITC-Dextran permeability. Results show that PPARγ agonist blocked high glucose-mediated activation of NFκB, while PPARγ antagonist aggravated it (Figure 7A). Additionally, PPARγ agonist protected against FITC-Dextran permeability elevation promoted by hyperglycemic condition whereas PPARγ antagonist exacerbated it (Figure 7B). This suggests the protective role of PPARγ against the inflammatory and permeability alterations induced by hyperglycemic condition on cerebral endothelial cells. Interestingly, all polyphenol-rich plant extracts as well as quercetin, caffeic and chlorogenic acids significantly protected against *PPARγ* gene expression down-regulation caused by hyperglycemic condition (Figure 7C). 

### 2.5. Detection of Polyphenols at the Intracellular Level or Membrane-Bound to Cerebral Endothelial Cells in Normoglycemic or Hyperglycemic Condition

There is still a lack of data regarding the molecular mechanisms involved in the uptake of polyphenols by cells. Both Pgp and BCRP are main efflux transporters regulating the BBB paracellular permeability. To determine whether these transporters could be implicated in the interaction between polyphenols and cerebral endothelial cells, bEnd3 cells were exposed to pure quercetin, caffeic, chlorogenic or gallic acids in normoglycemic or hyperglycemic condition, in the presence or not of Pgp or BCRP specific inhibitor. Then, polyphenols present at the intracellular level or membrane-bound to cells were extracted and identified by mass spectrometry. Data show that quercetin, its methylated metabolite isorhamnetin produced by cells and caffeic acid were present at the intracellular level while chlorogenic and gallic acids were not detected in cells (Table 1). The methylated metabolite of caffeic acid, namely ferulic acid, was targeted during mass spectrometry analysis but not detected. Intracellular caffeic acid concentrations ranging 2 nM in normoglycemic condition were not significantly different during high glucose condition, in the presence or not of Pgp and BCRP inhibitors. Intracellular levels of quercetin reaching 15 nM in normoglycemic condition were also not significantly changed in hyperglycemic condition. However, they were elevated in the presence of BCRP inhibitor in normoglycemic or hyperglycemic condition. Similarly, intracellular isorhamnetin concentrations depicted 3 to 5-fold higher than those of parent quercetin were increased in the presence of BCRP inhibitor during normoglycemic or hyperglycemic condition. The presence of Pgp inhibitor did not modulate intracellular levels of caffeic acid, quercetin and isorhamnetin. These data suggest a higher accumulation of both flavonoids quercetin and isorhamnetin in cells during BCRP inhibition and thus an involvement of BCRP in their efflux. Furthermore, all polyphenols were detected membrane-bound to cerebral endothelial cells. Concerning caffeic acid, its membrane-bound concentrations were 10-fold higher that those measured from the intracellular compartment and significantly reduced by the hyperglycemic condition, in the presence or not of Pgp and BCRP inhibitors. A similar decrease in the membrane-bound levels of chlorogenic and gallic acids was depicted during hyperglycemic condition, in the presence or not of Pgp and BCRP inhibitors. Conversely, membrane-bound concentrations of quercetin and isorhamnetin were not changed by the hyperglycemic condition, in the presence or not of Pgp and BCRP inhibitors. Altogether, these findings raise the possibility that high glucose condition attenuated the interaction of the phenolic acids caffeic, chlorogenic and gallic acids with cerebral endothelial cells, independently of Pgp and BCRP efflux transporters. In parallel, the hyperglycemic condition did not impact the interaction of cells with the flavonoids quercetin and its methylated metabolite isorhamnetin, despite the fact that their efflux may involve a BCRP transporter. 

## 3. Discussion

Hyperglycemia has been reported to alter the function of cerebral endothelial cells from the BBB, aggravating the risk of cerebrovascular complications such as stroke during diabetes [22,23]. There is still a lack of therapeutic strategies aiming to improve hyperglycemia-induced cerebral endothelial dysfunctions. This study provides evidence for the protective effect of polyphenols on inflammatory and permeability markers on murine bEnd3 cerebral endothelial cells exposed to hyperglycemic condition. Our choice of the immortalized bEnd3 cell line is in line with literature data reporting its suitability for a mimetic system of the BBB permeability, with respect to the production of tight junction proteins and a variety of transporters including the efflux transporters Pgp and BCRP as well as glucose transporter type 1 (GLUT-1) [24,25]. The experimental hyperglycemia was mimicked by a high glucose concentration (33 mM) and conducted in a time-dependent manner from 3–24 h, given that it caused time-dependent oxidative stress and deregulation of vasoactive markers without affecting the viability of bEnd3 cells in our previous studies [20,26]. Four characterized polyphenol-rich extracts from the French medicinal plants *A. borbonica*, *A. triplinervis*, *D. viscosa* and *T. bentzoe*, and their predominant polyphenols quercetin, caffeic, chlorogenic and gallic acids were assessed, since we previously showed that they exhibited antioxidant properties on bEnd3 cells during hyperglycemic condition [21]. These polyphenolic plant extracts were prepared from the leaves which constitute the most traditionally collected plant compartments used for herbal remedies [27], despite the fact that here a pharmacological approach was achieved by using aqueous-acetonic extraction to enrich polyphenolic fractions [21]. Accordingly, a pharmacological dose of 10 µM of polyphenols was used. This dose is broadly reported in the literature [28]. Even if it is considered higher than circulating levels of polyphenols close to 1 µM in nutritional studies [29], our published data indicated an absence of cytotoxic action on bEnd3 cells in our experimental conditions [21]. Thus, the biological effects of polyphenols observed in the present study could not be related to an alteration of the cellular viability. 

First, our results demonstrate that the hyperglycemic condition caused inflammatory alterations associated with monocyte adhesion and transendothelial migration, and the anti-inflammatory action of polyphenols. More particularly, high glucose induced a pro-inflammatory response through an increased NFκB transcriptional activity, an altered expression of genes coding for IL-1β, IL-6, TNF-α, IL-10, COX-2 and iNOS. These findings are consistent with literature data showing that diabetes/hyperglycemia-related vascular injury involves the activation of the transcriptional factor NFκB as a pivotal dynamic system initiating the production of pro-inflammatory mediators [6,30,31]. Likewise, several literature data reported the deleterious action of hyperglycemia on the activation of NFκB and pro-inflammatory cytokines production in endothelial cells, despite cerebral endothelial cells remaining poorly studied [30,31,32]. Besides the control of cytokines synthesis, NFκB enhances the production of enzymes like COX-2 and iNOS which generate mediators of inflammation. Our results indicate that high glucose condition increased *COX-2* and *iNOS* gene expression in cerebral endothelial cells. This is consistent with published data reporting hyperglycemia-induced NFκB nuclear translocation and up-regulation of COX-2 and iNOS in endothelial cells [31,33]. While COX-2 produces arachidonic acid-derived prostaglandin E2 known to maintain the inflammatory activation of the endothelium, iNOS promotes nitric oxide synthesis which contributes to oxidative stress and endothelial nitric oxide synthase uncoupling, reducing vasodilation [34]. Aljofan and Ding [35] demonstrated that hyperglycemia provokes oxidative stress through ROS overproduction in microvascular endothelial cells. Specific inhibition of COX-2 abolishes this deleterious action of hyperglycemia. Xing and co-workers [36] reported the activation of NFκB-dependent inflammatory pathway with higher levels of IL-1β, IL-6, TNF-α as well as iNOS and COX-2 activation in the aorta of streptozotocin-induced diabetic rats. Interestingly, pre-treatment of diabetic rats with the antioxidant flavonoid troxerutin reduces the diabetes-mediated elevation of both iNOS and COX-2 as well as cytokines production via NFκB inhibition. Other families of polyphenols such as anthocyanins and phenolic acids were found to alleviate endothelial dysfunctions by exerting antioxidant, anti-inflammatory and vasorelaxant effects [17,18,32,37,38,39]. Similarly, we recently reported that hyperglycemia-mediated oxidative stress in bEnd3 cerebral endothelial cells is prevented by a pharmacological inhibitor of NFκB or polyphenolic extracts from *A. borbonica*, *A. triplinervis*, *D. viscosa* and *T. bentzoe* as well as pure quercetin, caffeic, chlorogenic and gallic acids [21]. This may contribute to explaining here why such polyphenols exhibited protective properties by reducing pro-inflammatory alterations caused by hyperglycemic condition. Mechanistically, it was found that polyphenols such as quercetin, caffeic and chlorogenic acids acting as ROS scavengers counteract NFκB nuclear translocation by inhibiting the phosphorylation of the inhibitor protein of NFκB (IκB) in human umbilical endothelial cells exposed to hyperglycemia [40] and in ex vivo aortic vessel model exposed to oxidative stress [17]. Moreover, the anti-inflammatory benefits of polyphenols may result from their ability to activate the redox-sensitive nuclear factor erythroid 2-related factor 2 (Nrf2) that itself down-regulates NFκB and cytokines gene expression [41]. Concordantly, our published studies provided evidence for the capacity of the polyphenol-rich plant extracts and pure polyphenols tested here, to improve Nrf2 production altered in in vitro and in vivo models of inflammatory conditions mediated by hyperglycemia or bacterial lipopolysaccharides [20,21,42]. These results emphasize the high biological interest of antioxidants such as polyphenols to block the critical regulatory loop between oxidative stress and inflammation well documented in literature [43].

The present study demonstrates that high glucose condition led to a higher MCP-1 secretion and an up-regulated *E-selectin* gene expression in cerebral endothelial cells. Concordantly, we found that monocyte adhesion to cerebral endothelial cells and transendothelial migration were enhanced in hyperglycemic condition. These results are in line with literature data showing that pro-inflammatory cytokines such as IL-1β and TNF-α activate the endothelium and promote the recruitment of monocytes to the site of injury via MCP-1 release and the production of adhesive molecules like E-selectin. If MCP-1 mediates the chemotaxis step, E-selectin is involved in the early steps of leukocyte recruitment at the endothelial surface, namely tethering and rolling. Then, these steps lead to leukocyte adhesion to the activated endothelium. Given that a raised synthesis of cell adhesion molecules is associated with atherosclerosis [44], there is an important interest to develop pharmacological strategies to attenuate adhesive molecules production in order to limit the process of atherosclerosis and related vascular damage. Several polyphenols have been reported to modulate leukocyte adhesion and transendothelial migration in experimental models of inflammation-mediated endothelial dysfunctions and atherosclerosis [16,18,19]. Nevertheless, polyphenol effect on monocyte recruitment by cerebral endothelial cells during hyperglycemia remains poorly investigated. For the first time, our results provide evidence for the capacity of the polyphenolic extracts from *A. borbonica*, *A. triplinervis*, *D. viscosa* and *T. bentzoe* plants to reduce monocyte adhesion on cerebral endothelial cells and transendothelial migration in hyperglycemic condition. This could be related to the ability of plant polyphenols to attenuate the high glucose-mediated pro-inflammatory response described above, and to decrease MCP-1 and E-selectin production. In accordance with literature data obtained for other polyphenolic families [16,18,32], quercetin, caffeic, chlorogenic and gallic acids were found to exert protective effects, and thus could contribute here to the bioactivity of the plant extracts that improved the inflammatory response of cells in a hyperglycemic condition. Our previous study led us to identify these polyphenols as main compounds in the medicinal plant extracts. Nevertheless, it is not excluded that other compounds we detected in the plants such as ellagic acid, ellagitannins or kaempferol may contribute to the bioactivity of the plant extracts, given that literature data reported their antioxidant and anti-inflammatory effects [21]. It could be of interest to complete this study after fractionation and purification of the bioactive principles from the plant extracts. 

Many studies have reported that hyperglycemia increases brain injury and the incidence of worse outcome in stroke [22,23]. Among the molecular mechanisms responsible for vascular damage and the BBB disruption in diabetes-associated hemorrhagic transformation in ischemic stroke, the deregulation of tight junctions is well documented. Occludin, claudins and ZO represent key tight junction proteins regulating the paracellular permeability. Our study demonstrates that hyperglycemic condition increased the permeability to FITC-Dextran marker and decreased the production of occludin, claudin-5, ZO-1 and ZO-2 tight junctions in cerebral endothelial cells. Consistently, literature data reported the deleterious action of diabetes/hyperglycemia on the BBB permeability damage associated with diminished tight junctions on in vitro and in vivo models of stroke. Consequently, antioxidant and anti-inflammatory molecules including the polyphenols quercetin, caffeic, chlorogenic and gallic acids were reported as relevant pharmacological strategies helping to improve the BBB integrity, despite the fact that their action has been poorly investigated in the diabetic condition [39,45,46,47]. In an innovative way, our study highlights the capacity of the polyphenolic plant extracts and pure polyphenols tested to counteract high glucose deleterious action on cerebral endothelial cells, by decreasing FITC-Dextran permeability and improving tight junctions production. Such protective effects of polyphenols could be attributed to their ability described above to decrease the activation of NFκB, the production of cytokines and monocyte recruitment in hyperglycemic condition, and thus to attenuate the endothelial dysfunction and permeability damage. Importantly, our study demonstrates that PPARγ agonist decreased high glucose-mediated NFκB activation and permeability alteration, while PPARγ antagonist aggravated them. This result suggests the protective action of PPARγ and agrees with literature data reporting the key role of PPARγ in the protection of the cerebrovascular endothelium during stroke [48]. While PPARγ knockdown is associated with a higher cerebral infarct volume [49], PPARγ activation reduces the death of ischemic neurons by inhibiting oxidative stress [50]. Moreover, PPARγ pathway may improve stroke outcomes in the diabetic condition by decreasing neuroinflammation via NFκB inhibition and preserving the BBB integrity via tight junctions production [6,7]. PPARγ was also cited as a possible therapeutic target for the treatment of cerebral ischemia/reperfusion injury [6,51]. Interestingly, our findings demonstrate that the four polyphenolic medicinal plant extracts as well as quercetin, caffeic and chlorogenic acids improved PPARγ production that was lowered by high glucose. Such a positive regulation of PPARγ by these polyphenols may constitute a novel mechanism of action helping to understand their protective effects against inflammatory and permeability alterations of cerebral endothelial cells in the hyperglycemic condition. Further studies will be needed to assess polyphenol action during PPARγ inactivation and to precise their link with PPARγ pathway regulation. 

Noticeably, the present study underlines the possibility of a differential bioactivity extent of polyphenols on inflammatory and permeability markers, depending on the nature of the plant extract, phenolic compound and molecular target tested. This may be related to the differential complexity of the plant matrix with possible synergistic actions of phytochemicals. Moreover, polyphenol structure-activity relationships were particularly documented for the antioxidant capacity of flavonoids and phenolic acids [52]. The differential bioactivity extent of polyphenols may also result from a differential cellular accessibility rate or a differential interaction with cerebral endothelial cells. To assess polyphenol uptake in cells treated with pure quercetin, caffeic, chlorogenic or gallic acids, we compared the intracellular and membrane-bound levels of polyphenols in normoglycemic or hyperglycemic condition, in the presence or not of inhibitors of main Pgp and BCRP efflux transporters. Quercetin, caffeic, chlorogenic and gallic acids were detected membrane-bound to cells, while only caffeic acid, quercetin and its methylated metabolite isorhamnetin produced by cells were present in cells. The flavonoid quercetin, characterized by a more lipophilic structure than phenolic acids like caffeic acid [15], was detected at intracellular levels 10-fold higher than those of caffeic acid. This suggests the capacity of quercetin to cross the cellular membrane more easily than the phenolic acids tested and thus structure-uptake relationships. The low intracellular concentration of caffeic acid could account for the absence or undetectable level of ferulic acid, expected as a major methylated metabolite of caffeic acid [29]. We previously detected caffeic and ferulic acids in the infarcted cerebral hemisphere of mice intraperitoneally-treated with caffeic acid or *A. borbonica* plant extract, during an experimental stroke combined with hyperglycemia [20]. Other authors depicted phenolic acids including caffeic and gallic acids [53] as well as flavonoids such as quercetin [54] at nanomolar concentrations in the brain, providing evidence for their capacity to pass through the BBB. Based on cellular models, polyphenol uptake was found to be time-dependent [55] and limited for esterified molecules like chlorogenic acid due to the esterification of caffeic acid moiety composing chlorogenic acid [56]. This may explain here why chlorogenic acid was undetected in cerebral endothelial cells. To our best knowledge, our findings show for the first time that hyperglycemic condition attenuated the interaction of the phenolic acids caffeic, chlorogenic and gallic acids with cerebral endothelial cells, independently of Pgp and BCRP efflux transporters. Further studies will be needed to decipher the molecular mechanisms/possible membrane receptors induced by these phenolic acids and that could be deregulated in hyperglycemic condition. In parallel, our data indicate that the hyperglycemic condition did not impact the interaction of cells with the flavonoids quercetin and isorhamnetin, despite the fact that their efflux may involve BCRP transporter. Functional BCRP has been detected in bEnd3 cerebral endothelial cells and reported to regulate the paracellular tightness [8]. According to literature data, quercetin and isorhamnetin also exert inhibitory effects on BCRP efflux function and may be candidates for potentiating drug delivery in the brain [57,58]. The detection of isorhamnetin, known to originate from the methylation of quercetin [29], shows the capacity of cerebral endothelial cells to metabolize quercetin in normoglycemic or hyperglycemic condition, and suggests the possible contribution of isorhamnetin to the biological effects of quercetin. Consistent with vascular protection, isorhamnetin was shown to protect human brain microvascular endothelial cells from oxidative stress-mediated NFκB activation and apoptosis [59], and to improve hyperglycemia and lipid peroxidation in streptozotocin-induced diabetic rats [60]. It will be important to assess the biological effects of the polyphenol-rich medicinal plant extracts in an in vivo condition, in order to better consider the metabolic fate of polyphenols and evaluate their potential as promising reagents for improving hyperglycemia and ischemia-induced cerebrovascular damage.

## 4. Materials and Methods

### 4.1. Preparation and Characterization of Polyphenol-Rich Medicinal Plant Extracts

The medicinal plants collected in Réunion Island (France) and botanically identified at the University of Réunion Island with voucher number were *A. borbonica* (Rubiaceae, RUN052-F), *A. triplinervis* (Asteraceae, TCN-P061), *T. bentzoe* (Combretaceae, TCN-P009) and *D. viscosa* (Sapindaceae, TCN-P028). Plants declaration to the Nagoya protocol for Access and Benefit-Sharing Clearing-House was also performed (ABSCH-IRCC-FR-252879-1). Medicinal plant leaves were reduced to powder after airflow drying at 45 °C. Then, each plant powder (2 g) was dissolved in 10 mL of an aqueous-acetonic solution (70%, v/v, Sigma-Aldrich, St-Louis, MO, USA). The mixture obtained was incubated at 4 °C for 90 min, centrifuged at 1400 g at 4 °C for 20 min. Polyphenol-rich supernatants were collected and stored at −80 °C until analysis. According to the method previously published [42], an ultra-performance liquid chromatography coupled to electrospray ionization-tandem mass spectrometry analysis (UPLC-ESI-MS-MS, Agilent Technologies, Les Ulis, France) was achieved to identify polyphenols present in the medicinal plant extracts. The medicinal plants *A. triplinervis*, *D. viscosa*, *A. borbonica* and *T. bentzoe* were found to exhibit total polyphenol contents reaching 0.6, 3.2, 3.4 and 8.1 g gallic acid equivalent (GAE)/100 g of plants, respectively. Caffeic acid, chlorogenic acid and quercetin derivatives were detected as main polyphenols present in *A. borbonica*, *A. triplinervis* and *D. viscosa* plants, while gallic acid derivatives were detected in *T. bentzoe* plant [21]. Consistently, pure quercetin, caffeic, chlorogenic and gallic acids were used as control polyphenols in the experiments, and these chemical compounds were purchased from Sigma Aldrich (St-Louis, MO, USA). 

### 4.2. Cell Culture

Murine bEnd3 cerebral endothelial cells (ATCC^®^ CRL-2299™, Manassas, VA, USA) and human THP-1 monocytic cells (ATCC^®^ TIB-202™, Manassas, VA, USA) were cultured in Dulbecco’s Modified Eagle Medium (DMEM) containing 25 mM glucose, 10% heat-inactivated fetal bovine serum, 5 mM L-glutamine, 50 µU/mL penicillin and 2 µg/mL streptomycin (Pan Biotech, Dutscher, Brumath, France). A transfection of bEnd3 cells with the plasmid pNiFty-secreted alkaline phosphatase (pNiFty2-SEAP, InvivoGen, Toulouse, France) was performed to produce bEnd3-blue cell line model using lipofectamine 3000 (ThermoFischer Scientific, Les Ulis, France) and selection with Zeocin™ at 200 µg/mL (InvivoGen, Toulouse, France) over 3 weeks, according to the previously published method [20]. Five specific NFκB repeated transcription factor binding sites, an endothelial cell-leukocyte adhesion molecule proximal promoter and a SEAP reporter gene were contained on the pNiFty2-SEAP plasmid. bEnd3-blue cells were cultured in complete DMEM containing 200 µg/mL of Zeocin™. Cells were placed in an incubator at 37 °C and 5% of CO_2_.

### 4.3. Evaluation of NFκB Activation

bEnd3-blue cells expressing NFκB/SEAP reporter gene were cultured in a 96-well plate (1.8 × 10^4^ cells/well) in DMEM containing 5.5 mM of glucose (normoglycemic condition) for 24 h. Next, the medium was removed and cells were treated for 3, 8 or 24 h with each polyphenol-rich plant extract (10 µM GAE) or with a pure polyphenol, namely quercetin, caffeic, chlorogenic or gallic acids (10 μM) in hyperglycemic condition (33 mM glucose) or in normoglycemic condition (5.5 mM glucose). NFκB activation was evaluated through the measurement of NFκB/SEAP activity by using Quanti-Blue assay (InvivoGen, Toulouse, France), followed by absorbance measurement at 620–655 nm (FLUOstar Omega, Bmg Labtech, Cambridge, UK). For NFκB activation measurement in cells which were exposed to PPARγ agonist or antagonist, cells were preincubated with 10 μM of PPARγ agonist (Pioglitazone, Sigma Aldrich, St-Louis, MO, USA) or PPARγ antagonist (GW9662, Sigma Aldrich, St-Louis, MO, USA) in a humidified atmosphere (5% of CO_2_, 37 °C) for 1 h in normoglycemic condition. Then, cells were co-treated with PPARγ agonist or antagonist (10 μM) and high glucose level for 8 h. As described above, NFκB/SEAP activity was determined by Quanti-Blue assay and fluorescence measurement. 

### 4.4. Evaluation of Relative Gene Expression 

bEnd3 cells were cultured in a 6-well plate (3.5 × 10^5^ cells/well) in DMEM containing 5.5 mM of glucose (normoglycemic condition) for 24 h. Next, the medium was removed and cells were treated for 3 h with each polyphenol-rich plant extract (10 µM GAE) or with a pure polyphenol, namely quercetin, caffeic, chlorogenic or gallic acids (10 μM) in hyperglycemic condition (33 mM glucose) or in normoglycemic condition (5.5 mM glucose). An isolation of total RNA was performed with TRIzol™ (Invitrogen, ThermoFisher Scientific, Dardilly, France) and 4 µg of RNA were reverse-transcribed (RT) using Random hexamer primers (Eurogentec, Liège, Belgium) with SuperscriptTM II (Invitrogen, ThermoFisher Scientific, Dardilly, France). Subsequently, the quantitative polymerase chain reaction (qPCR) was achieved using fast SYBR green™ master mix (Applied Biosystems, ThermoFisher Scientific, Dardilly, France). cDNA fragment was amplified by PCR using the following specific primers: *claudin-5*: GCT-GGC-GCT-GGT-GGC-ACT-CTT-TGT (forward), GGC-GAA-CCA-GCA-GAG-CGG-CAC (reverse); *cyclooxygenase-2* (*COX-2*): TTT-GTT-GAG-TCA-TTC-ACC-AGA-CAG-AT (forward), CAG-TAT-TGA-GGA-GAA-CAG-ATG-GGA-TT (reverse); *E-selectin*: TCT-GGA-CCT-TTC-CAA-AAT-GG (forward), TGC-AAG-CTA-AAG-CCC-TCA-TT (reverse); *glyceraldehyde-3-phosphate dehydrogenase* (*GAPDH*): TTC-ACC-ACC-ATG-GAG-AAG-GC (forward), GGC-ATG-GAC-TGT-GGT-CAT-GA (reverse); *IL-1β*: GAC-CTT-CCA-GGA-TGA-GGA-CA (forward), AGC-TCA-TAT-GGG-TCC-GAC-AG (reverse); *IL-6*: CAA-GAG-ACT-TCC-ATC-CAG-TTG-C (forward), TTG-CCG-AGT-AGA-TCT-CAA-AGT-GAC (reverse); *IL-10*: ACC-TCC-TCC-ACT-GCC-TTG-CT (forward), GGT-TGC-CAA-GCC-TTA-TCG-GA (reverse); *inducible nitric oxide synthase* (*iNOS*): GCA-GCC-TGT-GAG-ACC-TTT-G (forward), GCA-TTG-GAA-GTG-AAG-CGT-TTC (reverse); *occludin*: CAC-ACT-TGC-TTG-GGA-CAG-AGG (forward), TGA-GCC-GTA-CAT-AGA-TCC-AGA-AG (reverse); *PPARγ*: AAA-CTC-TGG-GAG-ATT-CTC-CT (forward), TGG-CAT-CTC-TGT-GTC-AAC (reverse); *TNF-α*: CTT-CTG-TCT-ACT-GAA-CTT-CGG-G (forward), CAG-GCT-TGT-CAC-TCG-AAT-TTT-G (reverse); *ZO-1*: GCT-GTC-CCT-GTG-AGT-CCT-TC (forward), TGC-CAG-GTT-TTA-GGG-TCA-CA (reverse); *ZO-2*: AGA-AGA-ACC-TCC-GCA-AGA-GC (forward), GCC-TCA-CGG-TAT-TCA-ACC-GA (reverse). Raw data were analyzed using CFX Manager software (BioRad, Marnes-la-Coquette, France) and *GAPDH* gene expression was used for the normalization of target genes, according to the 2^−ΔΔC^_T_ method [61].

### 4.5. Quantification of Cytokine Secretion 

bEnd3-blue cells were cultured in a 6-well plate (3.5 × 10^5^ cells/well) in DMEM containing 5.5 mM glucose (normoglycemic condition) for 24 h. The medium was removed and cells were treated for 24 h with each polyphenol-rich plant extract (10 µM GAE) or with a pure polyphenol, namely quercetin, caffeic, chlorogenic or gallic acids (10 μM) in hyperglycemic condition (33 mM glucose) or in normoglycemic condition (5.5 mM glucose). Next, cell culture media were collected and analyzed using Mouse MCP-1 ELISA kit (eBioscience, ThermoFisher Scientific, Dardilly, France). Concerning the extraction of cellular proteins, 700 μL of phosphate buffer saline (PBS) were added to each well. After scrapping, cells were collected and centrifuged at 500 g for 4 min at 25 °C. Supernatants were removed and an addition of 200 μL of lysis buffer (Tris 25mM pH 8.3, KCl 10 mM, DTT 1 mM, EDTA 1 mM, Triton X-100 1%, protease inhibitors 1 X) was done. After resuspension of cell pellet in lysis buffer, a novel centrifugation was achieved at 500 g for 4 min at 25 °C, and supernatants containing proteins were collected. Total protein contents were determined using the bicinchoninic acid assay (BCA) [62]. 

### 4.6. Evaluation of Endothelial Permeability

bEnd3 cells were cultured in a 24-cell culture inserts (1 × 10^5^ cells/insert, 0.4 µm pore size Millicell^®^ Cell Culture Inserts, Merck Millipore, Guyancourt, France) in DMEM containing 5.5 mM glucose (normoglycemic condition) during 24 h. At confluence, the medium was removed and cells were treated for 24 h with each polyphenol-rich plant extract (10 µM GAE) or with a pure polyphenol, namely quercetin, caffeic, chlorogenic or gallic acids (10 μM) in hyperglycemic condition (33 mM glucose) or in normoglycemic condition (5.5 mM glucose). Then, cells were rinsed with PBS and Fluorescein IsoThioCyanate-Dextran (FITC-Dextran, 4 kDa, Sigma-Aldrich, St-Louis, MO, USA) diluted in Roswell Park Memorial Institute Medium 1640 (RPMI, Pan Biotech, Dutscher, Brumath, France) was added to the inner chamber, while RPMI medium without FITC-Dextran was added to the outer chamber. In order to determine the cellular permeability, the quantity of FITC-Dextran that has crossed was measured every 15 min for 1 h at an excitation wavelength of 492 nm and an emission wavelength of 520 nm (FLUOstar Optima, Bmg Labtech, Cambridge, UK). For endothelial permeability measurement in cells which were exposed to PPARγ agonist or antagonist, cells were pre-incubated with 10 μM of PPARγ agonits (Pioglitazone, Sigma Aldrich, St-Louis, MO, USA) or PPARγ antagonist (GW9662, Sigma Aldrich, St-Louis, MO, USA) in a humidified atmosphere (5% of CO_2_, 37 °C) for 1 h in normoglycemic condition. Then, cells were exposed to PPARγ agonist or antagonist (10 μM) in hyperglycemic condition for 24 h. The following steps were performed as described above, and fluorescence was measured at the excitation and emission wavelengths of 492 and 520 nm, respectively (FLUOstar Optima, Bmg Labtech, Cambridge, UK). The relative endothelial permeability was expressed as the percentage of the control normoglycemic condition. 

### 4.7. Evaluation of Monocyte Adhesion on Cerebral Endothelial Cells

The rate of monocyte adhesion was assessed by measuring the oxidation of the fluorogenic probe 2’,7’-bis-(2-carboxyethyl)-5-(and-6)-carboxyfluorescein, acetoxymethyl ester (BCECF-AM, ThermoFisher Scientific, Dardilly, France), according to the published method slightly modified [63]. Briefly, bEnd3 cells were cultured in a 96-well plate (1.8 × 10^4^ cells/well) in DMEM containing 5.5 mM glucose (normoglycemic condition) during 24 h. Next, the medium was removed and cells were treated for 3, 8 and 24 h with each polyphenol-rich plant extract (10 µM GAE) or with a pure polyphenol, namely quercetin, caffeic, chlorogenic or gallic acids (10 μM) in hyperglycemic condition (33 mM glucose) or in normoglycemic condition (5.5 mM glucose). Then, bEnd3 cells were rinsed twice with PBS and a first measurement of fluorescence was performed (Fb: unlabeled fluorescence) at an excitation and emission wavelengths of 492 and 520 nm, respectively (FLUOstar Optima, Bmg Labtech, Cambridge, UK). Then, THP-1 cells were incubated for 15 min with BCECF-AM fluorescent probe (20 µM in Hanks’ Balanced Salt Solution (HBSS) medium, Pan Biotech, Dutscher, Brumath, France). After a centrifugation at 400 g for 5 min at 25 °C, 100 µL of a 10^5^ fluorescent THP-1/mL cell suspension in RPMI medium were added. A second measurement of fluorescence was performed (Ft: total fluorescence) and cells were further co-incubated in a humidified atmosphere (5% of CO_2_, 37 °C), for 1 h. Next, non-adhering THP-1 cells were washed away using HBSS, and a measurement of the intensity of fluorescent adherent THP-1 cells was achieved (Fr: remaining fluorescence). The relative monocyte adhesion was expressed as the percentage of the control normoglycemic condition, after calculating the monocyte adhesion according to the following formula:

$$\mathrm{Adhesion}\;\left(\%\right)=\frac{\mathrm{Fr}\;-\;\mathrm{Fb}}{\mathrm{Ft}\;-\;\mathrm{Fb}}\;\times \;100$$

### 4.8. Evaluation of Monocyte Transendothelial Migration

bEnd3 cells were cultured in 12-cell culture inserts (12 × 10^4^ cells/insert, 0.4 µm pore size, Millicell^®^ Cell Culture Inserts, Merck Millipore, Guyancourt, France) in DMEM containing 5.5 mM glucose (normoglycemic condition) for 24 h. Next, at confluence, the medium was removed and cells were treated for 24 h with each polyphenol-rich plant extract (10 µM GAE) or with a pure polyphenol, namely quercetin, caffeic, chlorogenic or gallic acids (10 μM) in hyperglycemic condition (33 mM glucose) or in normoglycemic condition (5.5 mM glucose). Next, based on the Milenkovic et al. method [64] slightly modified, THP-1 cells were incubated for 15 min with BCECF-AM fluorescent probe (20 µM in HBSS medium, Pan Biotech, Dutscher, Brumath, France). bEnd3 cells were rinsed twice with PBS. A density of 5 x 10^4^ fluorescent THP-1 cells in 100 µL final volume of RPMI (Pan Biotech, Dutscher, Brumath, France) was added to the upper chamber and a solution of 10 ng/mL of MCP-1 (Standard Mouse MCP-1 recombinant protein, eBioscience, ThermoFisher Scientific, Dardilly, France) in RPMI was added in the lower chamber, for a co-incubation of 3 h in a humidified atmosphere (5% of CO_2_, 37 °C). The intensity of fluorescent THP-1 cells crossing bEnd3 cells was measured in the lower chamber at an excitation wavelength of 492 nm and an emission wavelength of 520 nm (FLUOstar Optima, Bmg Labtech, Cambridge, UK). The relative monocyte transendothelial migration was expressed as the percentage of the control normoglycemic condition.

### 4.9. Evaluation of Polyphenols Uptake by Cerebral Endothelial Cells

bEnd3 cells were cultured in a 6-well plate (3.5 × 10^5^ cells/well) in DMEM containing 5.5 mM of glucose (normoglycemic condition) over 24 h. Then, the medium was removed and cells were preconditioned or not with 5 µM of one efflux transporter inhibitor targeting Pgp (Valspodar, Sigma-Aldrich, St-Louis, MO, USA) or BCRP (KO143, Sigma-Aldrich, St-Louis, MO, USA) in normoglycemic (5.5 mM glucose) or hyperglycemic condition (33 mM glucose). After 1 h, quercetin, caffeic, chlorogenic or gallic acids (10 µM) were added for 3 h. Next, cell culture medium was removed and 1 mL of PBS was added to rinse cells twice. On one hand, to collect polyphenols which were possibly membrane-bound to cells, 500 µL of 0.4% bovine serum albumin in PBS solution (w/v) were added and samples were stored in tubes during 45 min at 4 °C. An addition of 500 µL of methanol/HCl (200 mM) was performed in each tube to extract polyphenols from cellular components. One the other hand, to collect polyphenols at the intracellular level, 1 mL of methanol/HCl (200 mM) was added to detach cells with the help of a scraper. Samples were collected and stored in tubes over 45 min at 4 °C. Next, all samples originating from the membrane-bound fraction or from the intracellular compartment were spiked with 5 µL of the internal standard syringic acid (1 µM) and centrifuged at 14,000 g over 5 min at 4 °C. Supernatants containing polyphenols were analyzed by ultra-high performance liquid chromatography coupled with diode array detection and Heated Electrospray Ionization (HESI)-Orbitrap mass spectrometer (Q Exactive Plus, ThermoFisher Scientific, Les Ulis, France), according to the method we previously described [20]. Mass spectra were registered in full scan mode from m/z 100 to 1500 in negative ion mode. Polyphenols were identified according to their accurate mass, retention time and MS/MS analysis. Data were acquired by XCalibur 4.0 software (Thermo Fisher Scientific Inc., Les Ulis, France). For quantitation, a mix of standard solutions of quercetin, caffeic, ferulic, chlorogenic, gallic and syringic acids was prepared in methanol with concentrations ranging 0.005–10 μM. In parallel, two quality control samples were analyzed in duplicate within each batch, together with two blank samples containing methanol. The intensity of peaks was plotted against the corresponding standard concentrations to establish the calibration curves. Internal standard recovery was determined similarly. Calibration curves were built according to the method published [20]. 

### 4.10. Statistical Analysis

Experiments were performed in three independent experiments (cellular passages) and data were expressed as means ± SEM. Data normalization according to the control normoglycemic condition was performed when appropriate. Statistical analysis was performed by using analysis of variance (ANOVA), followed by the Bonferroni’s multiple comparison test. For data reported on the Figure 2 and Figure 5, two-way ANOVA was used when two experimental parameters were assessed, namely the cellular medium composition and the time of treatment. For other data, one-way ANOVA was used when one experimental parameter was assessed, namely the cellular medium composition. Significant differences were considered for a *p* value < 0.05 according to the Program GraphPad Prism (GraphPad Software, Inc., San Diego, CA, USA). 

## 5. Conclusions

This study demonstrates the protective effect of polyphenol-rich extracts from four French medicinal plants as well as quercetin, caffeic, chlorogenic and gallic acids on cerebral endothelial cells exposed to an experimental hyperglycemia. Polyphenols attenuated pro-inflammatory and permeability alterations, decreased monocyte recruitment and improved the deregulation of NFκB/PPARγ pathways caused by hyperglycemic condition. The bioactivity extent of polyphenols depended on the nature of the polyphenol-rich plant extract, phenolic compound and molecular target considered. Importantly, polyphenols were detected at the intracellular level or membrane-bound to cerebral endothelial cells, with evidence for BCRP efflux transporter involvement. Therefore, these findings highlight the capacity of polyphenols to protect cerebral endothelial cells in hyperglycemic condition, and their relevance for the development of innovative pharmacological strategies aiming to improve cerebrovascular injury and stroke recovery during diabetes.

## Figures and Tables

**Table 1:** Concentrations of polyphenols detected at the intracellular level or membrane-bound to cerebral endothelial cells.

Concentration (nM)	NG	NG + Pgpi	NG + BCRPi	HG	HG + Pgpi	HG + BCRPi
**Intracellular**						
Caffeic acid	2.11 ± 0.02	1.91 ± 0.14	1.91 ± 0.24	1.96 ± 0.19	1.65 ± 0.36	1.60 ± 0.15
Chlorogenic acid	nd	nd	nd	nd	nd	nd
Gallic acid	nd	nd	nd	nd	nd	nd
Quercetin	15.70 ± 1.14	18.68 ± 1.51	24.36 ± 1.70 **	17.36 ± 0.80	21.77 ± 0.08	39.00 ± 9.27 ^#^
Isorhamnetin	58.59 ± 7.92	57.22 ± 8.13	124.64 ± 13.09 **	65.22 ± 2.49	76.24 ± 9.55	158.49 ± 22.51 ^##^
**Membrane-bound**						
Caffeic acid	26.18 ± 2.74	21.06 ± 4.22	18.00 ± 2.67	14.25 ± 2.80 *	14.26 ± 1.96 *	17.74 ± 3.52 *
Chlorogenic acid	16.25 ± 0.94	12.38 ± 3.28	11.33 ± 1.86	7.70 ± 0.90 *	5.73 ± 0.26 *	9.06 ± 3.00 *
Gallic acid	30.03 ± 3.75	26.43 ± 8.81	28.57 ± 8.31	12.35 ± 2.55 *	13.11 ± 2.27 *	10.21 ± 2.17 *
Quercetin	11.13 ± 2.23	9.14 ± 3.16	7.00 ± 2.94	8.81 ± 1.81	8.08 ± 3.97	9.85 ± 4.50
Isorhamnetin	69.14 ± 18.60	57.74 ± 17.78	86.24 ± 9.08	46.17 ± 8.69	50.12 ± 17.01	76.52 ± 14.28

Cells were preconditioned or not with 5 µM of one efflux transporter inhibitor targeting Pgp (Pgpi) or BCRP (BCRPi) in normoglycemic (NG) or hyperglycemic (HG) condition. After 1 h, quercetin, caffeic acid, chlorogenic acid or gallic acid (10 µM) was added for 3 h. Next, the polyphenols present at the intracellular level or membrane-bound to cells were extracted and identified by mass spectrometry. Peak intensities were plotted against the corresponding standard concentrations used to build the calibration curves. Intracellular and membrane-bound concentrations of polyphenols measured were expressed as nM of quercetin, caffeic, chlorogenic or gallic acids. Isorhamnetin levels were expressed as nM of quercetin equivalent. Data are means ± SEM of three independent experiments (three cellular passages). *: *p* < 0.05 and **: *p* < 0.01 as compared to NG; ^#^: *p* < 0.05, ^##^: *p* < 0.01 as compared to HG. nd: not detected.

## Data Availability

The data that support the findings of this study are available from the corresponding author upon reasonable request.

## Abbreviations

*A. borbonica*	*Antirhea borbonica*
*A. triplinervis*	*Ayapana triplinervis*
BBB	blood-brain barrier
BCA	bicinchoninic acid
BCECF-AM	2′,7′-bis-(2-carboxyethyl)-5-(and-6)-carboxyfluorescein acetoxymethyl ester
BCRP	breast cancer resistance protein
bEnd3	brain endothelial cells
COX	cyclooxygenase
DMEM	Dulbecco’s Modified Eagle Medium
*D. viscosa*	*Dodonaea viscosa*
E-selectin	endothelial-leukocyte adhesion molecule
FITC	fluorescein isothiocyanate
GLUT-1	glucose transporter type 1
HBSS	Hanks’ balanced salt solution
HG	hyperglycemic condition
IκB	inhibitor protein of NFκB
IL	interleukin
iNOS	inducible nitric oxide synthase
MCP-1	monocyte chemoattractant protein 1
MMP	matrix metalloproteinase
NFκB	nuclear factor kappa B
NG	normoglycemic condition
Nrf2	nuclear factor erythroid 2-related factor 2
PBS	phosphate buffer saline
PPARγ	peroxisome proliferator-activated receptor gamma
Pgp	P-glycoprotein
RPMI	Roswell park memorial institute medium
SEAP	secreted alkaline phosphatase
*T. bentzoe*	*Terminalia bentzoe*
TNF-α	tumor necrosis factor alpha
UPLC-ESI-MS-MS	ultra-performance liquid chromatography coupled to electrospray ionization-tandem mass spectrometry
ZO	zona occludens
